# Shape Variation in Aterian Tanged Tools and the Origins of Projectile Technology: A Morphometric Perspective on Stone Tool Function

**DOI:** 10.1371/journal.pone.0029029

**Published:** 2011-12-27

**Authors:** Radu Iovita

**Affiliations:** Department of Palaeolithic Studies, Römisch-Germanisches Zentralmuseum Mainz, Schloss Monrepos, Neuwied, Germany; University of Oxford, United Kingdom

## Abstract

**Background:**

Recent findings suggest that the North African Middle Stone Age technocomplex known as the Aterian is both much older than previously assumed, and certainly associated with fossils exhibiting anatomically modern human morphology and behavior. The Aterian is defined by the presence of ‘tanged’ or ‘stemmed’ tools, which have been widely assumed to be among the earliest projectile weapon tips. The present study systematically investigates morphological variation in a large sample of Aterian tools to test the hypothesis that these tools were hafted and/or used as projectile weapons.

**Methodology/Principal Findings:**

Both classical morphometrics and Elliptical Fourier Analysis of tool outlines are used to show that the shape variation in the sample exhibits size-dependent patterns consistent with a reduction of the tools from the tip down, with the tang remaining intact. Additionally, the process of reduction led to increasing side-to-side asymmetries as the tools got smaller. Finally, a comparison of shape-change trajectories between Aterian tools and Late Paleolithic arrowheads from the North German site of Stellmoor reveal significant differences in terms of the amount and location of the variation.

**Conclusions/Significance:**

The patterns of size-dependent shape variation strongly support the functional hypothesis of Aterian tools as hafted knives or scrapers with alternating active edges, rather than as weapon tips. Nevertheless, the same morphological patterns are interpreted as one of the earliest evidences for a hafting modification, and for the successful combination of different raw materials (haft and stone tip) into one implement, in itself an important achievement in the evolution of hominin technologies.

## Introduction

**Figure 1:**
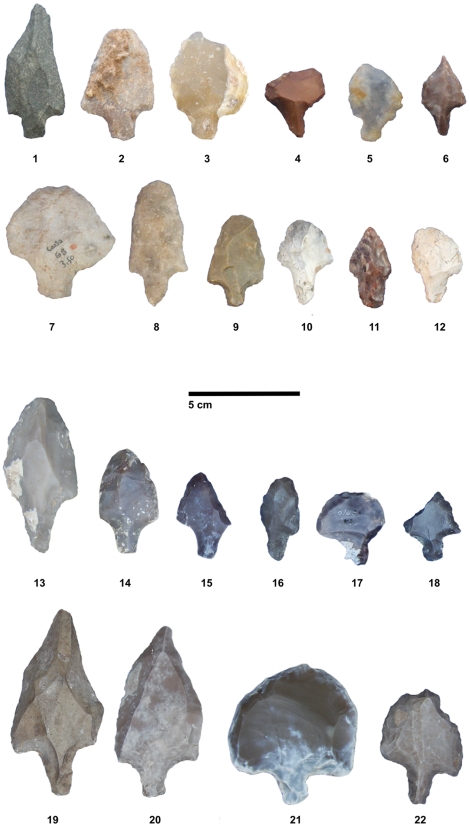
Some examples of Aterian tanged tools. Nos. 1–6 from the site of Contrebandiers, Morocco; 7–12 from the site of El-Mnasra, Morocco; 13–18 from the site of El-Oubira, Algeria; and 19–22 from the site of Oued Djouf, Algeria.

The ability of human hunters to ‘kill at a distance’ [1], [2] is often considered one of the hallmarks of modern human behavior. Such an ability embodies the cultural transcendence of the human body's condition with the aid of technology and has deep implications for the self-understanding of our species's uniqueness in the animal kingdom. For this reason, the search for evidence of projectile weapon technologies in the Stone Age has superseded the search for evidence of mere hunting activities, the latter having slid in the background of pre-human hominin behavioral repertoire [3]–[5]. Because ‘safe hunting’ is considered to have given anatomically-modern humans a competitive advantage against Neandertals during the last Out-of-Africa event (e.g., [6], [7]), it is extremely important to rigorously examine claims for the existence of such technologies, even when the superficial examination of the morphology of a particular tool suggests a clear functional determination. Such is the case of the Aterian tanged (or stemmed) point, a type of stone tool found throughout North Africa in a variety of ecological, geographical, and chronological contexts within the African Middle Stone Age (MSA), and which exhibits a simple form that is sometimes reminiscent of stemmed arrowheads or spear points from much later time periods (Figure 1).

**Figure 2:**
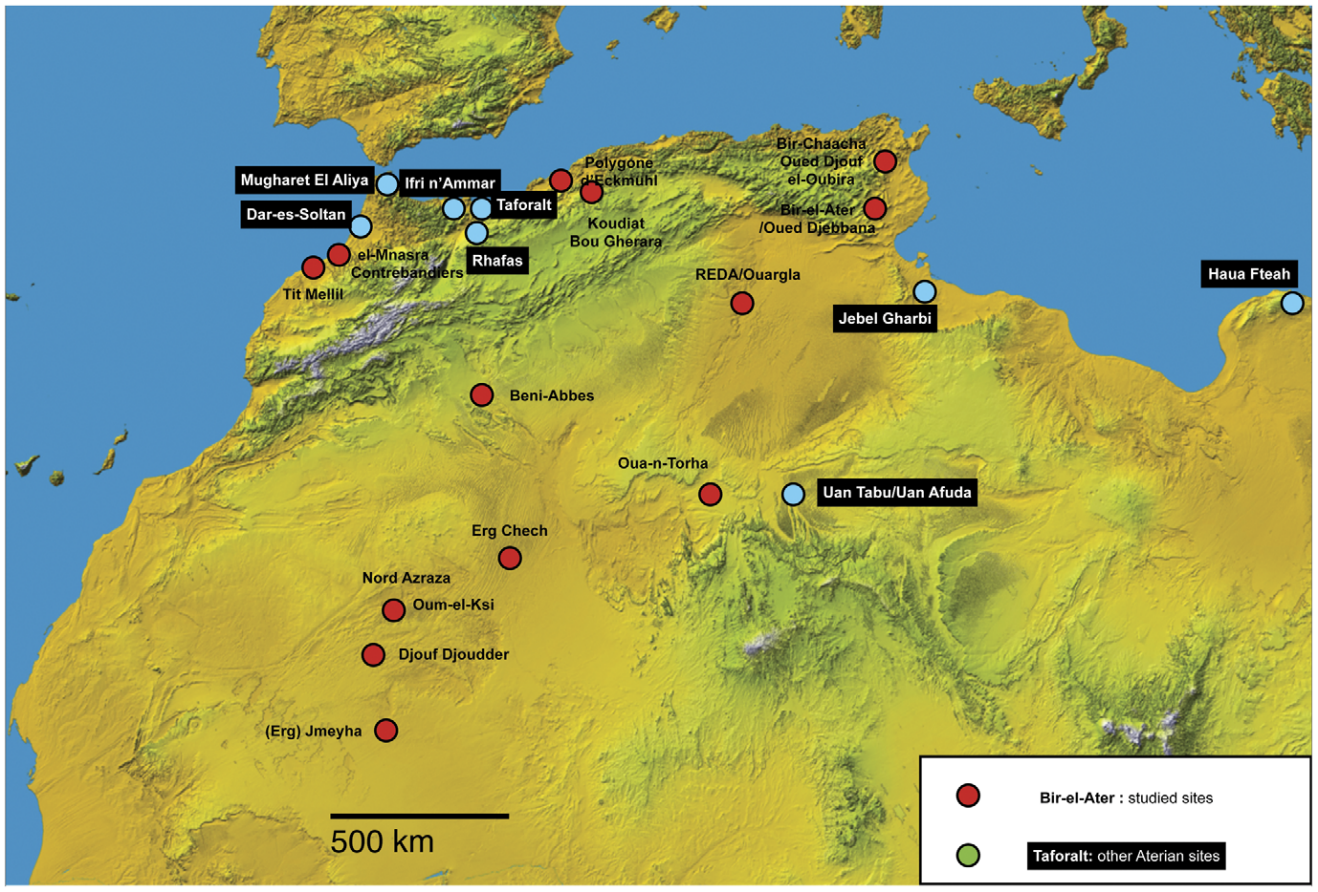
Map of the Aterian sites discussed in this article (red circles, see also **Table 1**), with other important Aterian sites shown by the green circles. Base map from NASA http://www2.jpl.nasa.gov/srtm/africa.htm.

The Aterian as a cultural entity [8]–[11] can be found across most of North Africa (see Figure 2) and is differentiated from the Mousterian and essentially defined by the presence of these tanged (or stemmed) tools. Largely because the tang has almost always been assumed to imply hafting (but see [12], [13]), but also arrows or at least spear-points, the Aterian was long considered to be technically advanced in comparison with the Mousterian, and was placed late in the Paleolithic sequence, between the latter and the Upper Paleolithic [9], [14]. Along with its association with anatomically modern human fossils [15], [16], the presumed successful combination of two raw materials (the stone point and the presumably wooden shaft) into a single composite tool has cemented some of the untested ideas about this technology and its implications for human evolution. Ecologically, it is generally seen as an adaptation to hunting with projectile weapons in open grassland to arid environments [17]–[19], and has been linked with the first Out-of-Africa expansion of modern humans through the Sahara and toward the Mediterranean coast [20].

Further, given the assumption that at least some of the tanged tools were projectile tips, the Aterian has been frequently incorporated into the wider phenomenon of MSA point styles (e.g., [17], [21], subsequent discussions limiting themselves to the determination of the launching technology [7], [22]. However, even assuming that they were indeed used for penetrative tasks, using ballistic parameters such as ‘tip cross-sectional area’ (TCSA) and ‘tip cross-sectional perimeter’ (TCSP) to distinguish between dart and arrow tips, Aterian tanged tools are known to fall just outside the accepted range for both flying projectiles [7], [22]. More importantly, aside from the tang, the morphology of the ‘tip’ of Aterian tools is very variable and, as such, lends itself to a classification similar to that of Mousterian tools in Europe and the Middle East. Recognizing this diversity led Tixier [23], [24] to propose a typology which creates functional, or at least functionally-inspired distinctions between edge-tools (such as scrapers) and penetrative tools (such as projectile points) on a purely morphological basis. However, as with the Bordian typology itself [25], the distinctions that form the basis of this classification system are arbitrary cutoffs within a shape continuum [26]–[28] which ranges from pointed and elongated triangular forms to rounded and squat blunt forms, as is demonstrated in the rest of this article. Therefore, any claim that Aterian tanged pieces represent an early projectile technology must be evaluated within an explanation of the continuous nature of the shape variation present in these tools.

The importance of correctly interpreting the function of Aterian stemmed tools is underlined by recent dating results, which suggests that, contrary to early assumptions, it could date to as early as MIS 5 and before [29]. More specifically, new dates from a series of sites, such Mugharet el-Aliya [30], Rhafas [31], Ifri n'Ammar [29], Dar-es-Soltan [32], and Contrebandiers [33] have demonstrated that tanged tools can be found in the earliest part of the North African Middle Stone Age, making them potentially the earliest evidence of prehistoric stone-tipped weaponry. However, the precise way in which they actually fit within a prehistoric technological system, including whether or not they were part of flying projectile armatures or thrusting spears, has never been rigorously determined, despite the crucial role that both projectiles and hafting are thought to play in the evolution of human cultural adaptations.

The determination of an object's function is best achieved through microscopic studies of use-wear traces, including the presence of use-related macro-level damage and of polishes that are revealed at high magnification (for a review of the method for projectile points see [34]). However, the labor costs associated with this method often result in very small sample sizes, a problem compounded by a lack of consensus among specialists regarding the experimental standards used to make the determinations ([35], [36], but see [37]–[40] for new quantitative approaches). Moreover, resharpening often removes the tool's active edges, resulting in the loss of information from exactly those tools which were most likely curated and re-used. Identifying broken lithics as having once been part of stone-tipped flying weapons is particularly fraught with problems of taphonomic and use-related equifinality, although recent studies have begun to quantify the extent and location of damage expected from such a use [41], [42]. Finally, and perhaps most importantly, large enough samples of Aterian stemmed pieces are best known from old excavations or surface collections in museums, with only a few recent excavations having yielded new samples, two of which are presented in this study (Contrebandiers and El-Mnasra, see Table 1). This leads to difficulties due to sometimes large differences in curation and conservation of the specimens, as well as the presence of patina and desert varnish, factors which render the secure attribution of damage to use-related activities impractical, or, at times, impossible.

**Table 1:** Assemblages used in this article. See also Figure 1 for site locations.

	n	Country	Location	Reference
**Excavated assemblages**				
Bir-Chaacha	51	Algeria	Musée de l'Homme, Paris	[8]
Bir-el-Ater	19	Algeria	Musée de l'Homme, Paris	[8]
*Contrebandiers*	33	Morocco	INSAP, Rabat	[98], [99]
*el-Mnasra*	20	Algeria	INSAP, Rabat	[100]
el-Oubira	97	Algeria	Musée de l'Homme, Paris	[10]
Oued Djouf el Djemel	140	Algeria	Musée de l'Homme, Paris	[101]
Tit Mellil	35	Morocco	MNAST, Rabat	[102]
Subtotal	395			
**Surface collections**				
Beni-Abbes	23	Algeria	Musée de l'Homme, Paris	
Djouf Djoudder	10	Mali	Musée de l'Homme, Paris	coll. Anstett
Erg Chech	9	Algeria	Musée de l'Homme, Paris	
[Erg] Jmeyha	4	Mali	Musée de l'Homme, Paris	coll. Anstett
Koudiat Bou Gherara	2	Algeria	Musée de l'Homme, Paris	[103]
Nord Azraza	3	Mali	Musée de l'Homme, Paris	coll. Anstett
Polygone d'Eckmuhl	16	Algeria	Musée de l'Homme, Paris	[104]
Oua-n-Torha	3	Algeria	Musée de l'Homme, Paris	coll. Monod
Oum-el-Ksi	4	Mali	Musée de l'Homme, Paris	coll. Anstett
RDAC/REDA (Ouargla)	20	Algeria	Musée de l'Homme, Paris	coll. Morel
Unknown North Africa	13	–––––	Musée de l'Homme, Paris	
Tebessa	5	Algeria	Musée de l'Homme, Paris	
Subtotal	112			
**TOTAL**	**507**			

**Figure 3:**
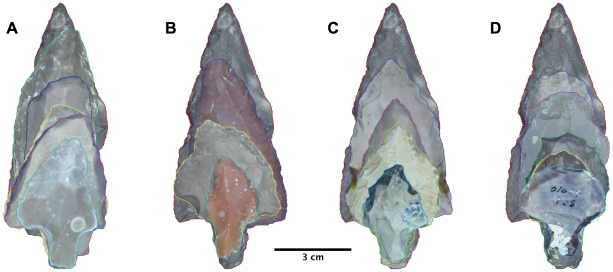
Theoretical resharpening trajectories of a single large, unretouched point from the site of Oued Djouf, Algeria. A. preferring the left lateral edge; B. preferring the right lateral edge; C. keeping a pointed end, resharpening both lateral edges symmetrically; D. resharpening the lateral edges into a blunted, rounded, endscraper-like tip.

In such cases, an alternative to microscopic methods of determining the function of a stone artifact is to examine changes in tool morphology, with the assumption that repeated repair cycles *of the active* part of the tool through resharpening will have recognizable, quantifiable effects. That morphological variability due to repeated retouch episodes is related to resharpening and that a sufficiently long sequence of tools preserves a ‘record’ of these steps has been demonstrated time and again in stone tools from Lower Paleolithic to Holocene contexts the world over ([43]–[55]). The recycling of weapon tips (be they lance-tips or flying projectile dart or arrow points), accompanied by a change in function, has also been documented [56]–[59]. A recent example of functional determination in unquestionably hafted North American bifaces, distinguishing between cutting and projectile tools based on resharpening patterns is provided by Harper and Andrefsky [60]. In the case of Aterian tanged tools, it is possible that several different types of repeated resharpening took place, as illustrated hypothetically in Figure 3, using a single large, unretouched point from the site of Oued Djouf as a starting point for different resharpening trajectories. Here, reduction strategies centered around the preservation of a single edge (A and B) or of two edges simultaneously, with (C) or without (D) the preservation of a point are presented using a mosaic of real tools from Aterian assemblages.

## Results

The analysis was carried out in two steps: first, the degree to which retooling affects morphological variation in Aterian stemmed tools was assessed, followed by an investigation of the functional implications of the documented resharpening-related shape changes, with particular regard to the difference between penetrative (weapon-like) and cutting/scraping (edge-based) tools.

### Aterian tools were resharpened in the haft

**Figure 4:**
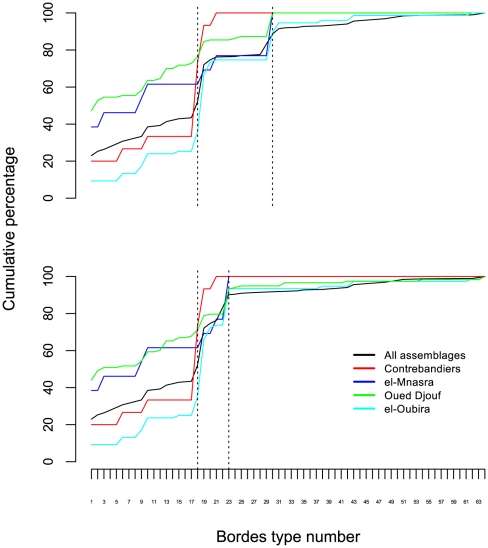
Cumulative graphs of Bordian types from the Aterian sample, showing similarity to typical Mousterian cumulative graphs, including the changes in the shape of the curve when the endscraper-tips (types 30–31) are re-classified as transverse scrapers (types 22–23).

As mentioned above, when the stem is ignored for classification purposes, the aggregate sample of Aterian tanged tools presented here (n = 507, see also Table 1 and Figure 2) exhibits some of the classic features of Middle Paleolithic European or Near Eastern assemblages, as the cumulative percentage graphs of the Bordian essential types shows (Figure 4). The difference between the two is the much greater frequency of endscrapers (types 30–31) in the Aterian sample, compared with a greater presence of sidescrapers (types 9–29) in typical Mousterian assemblages. This difference is, however, unlikely to reflect a real technological difference, since the principle behind the classification of unhafted retouched pieces relies on orienting them along the axis of flaking (the direction of removal of the blank from the core). When the tips of Aterian stemmed pieces are considered for a Bordian typology, these are necessarily oriented with the stem/tang down, regardless of the direction of flaking, resulting in a possible overrepresentation of ‘end-scrapers.’ In both cases, however, the presence of a ‘scraper’-rich component reveals a structure of morphological variation which is qualitatively similar to that present in Mousterian assemblages, where such variation has been shown to be due to repeated resharpening [27], [44].

**Figure 5:**
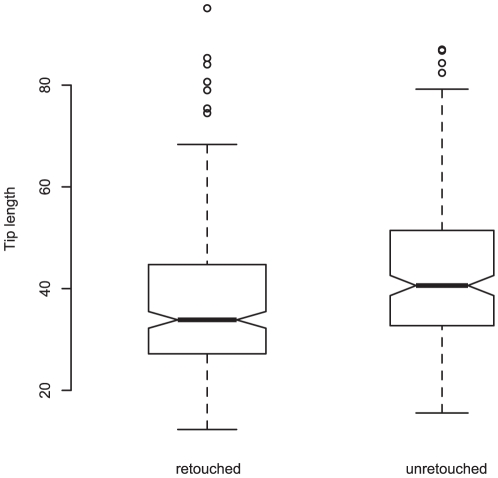
Tip lengths for retouched and unretouched Aterian tools, showing that reduction through retouch and resharpening affects this part of the tool.

Analyzing the variation in lengths of the tangs and the tips of the tools in the sample shows that the tip length of retouched pieces (types 6 and above) is significantly smaller than that of unretouched pieces (t (Welch 2-sample equal variance) = 7.5, df = 335, p<0.01, see also Figure 5). The difference is even greater between Levallois pieces (types 1–4) and scrapers (types 9–31). Meanwhile, the tang lengths themselves are similarly distributed between the retouched and the unretouched pieces (t (Welch 2-sample equal variance) = 0.8, df = 484.27, p = 0.42), supporting the hypothesis that resharpening through retouch is what is causing the reduction in size of the tips, while tang length varies randomly.

It has been recently proposed, on the basis of microscopic work on a sample of 11 Jebel Gharbi Aterian tools [13] that the two notches making up the tang were in fact the active part of the tool. This would, in part, explain the large variability of what has normally been interpreted as the ‘tip’ of the tools, which, under this assumption, would be free to vary. However, 87% of the 507 tangs were bifacially worked through a series of removals, rather than created by simple ventral or dorsal notching. This often led to the blunting of the edge of the tang notches by the creation of an abrupt intersection between the dorsal and ventral scars. Moreover, previous studies [61] have shown that, in Mousterian-type industries, the number of notches on a tool is a result of resharpening. Since resharpening does seem to affect the ‘tips,’ a certain variability with respect to the number of notches is also expected. However, the grand total of tanged tools possessing any notches beyond those that make up the tang is only 9, adding to the evidence that the function of the tang was indeed as a hafting modification, rather than the active part of the tool. Perhaps the traces found on the Jebel Gharbi tools are, in fact, hafting wear, rather than use-wear.

### Aterian tools were resharpened like cutting/scraping tools

**Figure 6:**
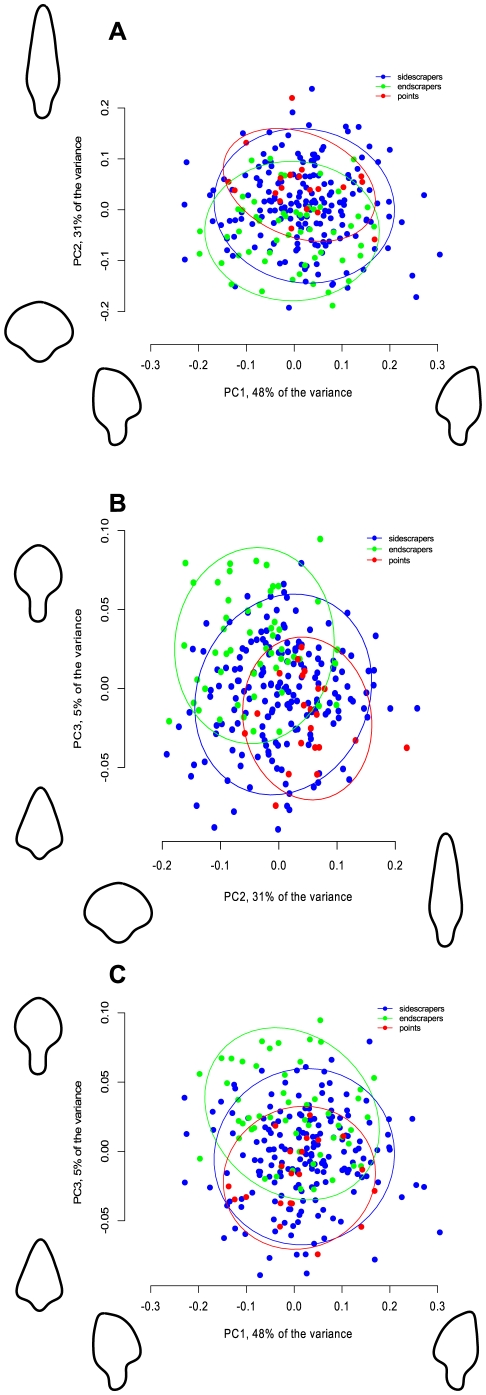
Graph showing the three main categories of tools (endscrapers, side-scrapers, and points) on pairwise-depicted axes of the first three principal components of shape variation. The outlines on each axis represent extreme shapes along each component (see Method for an explanation). The ellipses represent 95% confidence intervals around the centroid of each data cluster.

Having established that resharpening is taking place in the sample, and that it is primarily affecting the part of the tool traditionally known as the ‘tip,’ it is time to examine the nature of the morphological changes associated with resharpening. The first three principal components of the elliptical Fourier coefficients for the first 11 harmonics describing the size-free outline shapes in the aggregate Aterian sample account for 84% of the total variance. Visually, the first PC (48%) describes side-to-side asymmetry, the second PC (31%) shows elongation, and the third PC (5%) relates to relative tang length and tip roundness (see Figure 6). This is the case for the complete sample containing both retouched and unretouched pieces, with only small differences appearing when only retouched pieces are taken into consideration (PC 1 accounts for 46%, PC 2 for 30%, and PC 3 for 6% of the total variance in the sample containing only retouched pieces).

Plotting the Bordes tool types on the graphs of the PCs reveals that the differences between ‘points,’ (types 3, 4, and 6), ‘sidescrapers’ (types 9–29), and ‘endscrapers’ (types 30–31) are indeed of a continuous nature, with the 95% confidence ellipses overlapping in most cases (Figure 6). This shows that, while the different typological categories tend to cluster in the expected regions of the PC space (points are ‘pointy’ and endscrapers have round tips), there are no gaps in the data that justify a separation based on these subjective shape-evaluation criteria.

**Figure 7:**
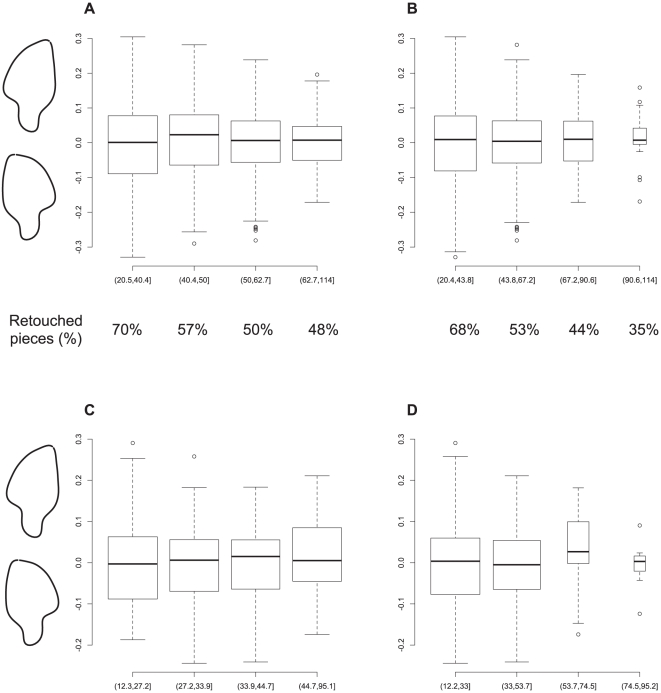
Boxplots showing the variation in PC 1 in the entire sample (A and B) and the retouched pieces only (C and D). A and C show length split in quantiles such that the samples are roughly equal, whereas B and D show the length split into four equal segments, resulting in uneven samples (shown also by the width of the boxes). For A and B, the percentage of retouched tools in each quartile are shown below.

Some of the shape variation is size-dependent. One of the most important patterns that emerges is that of a greater variation in PC1 values in the short tools (Figure 7). As mentioned before, this principal component has the visual equivalent of side-to-side asymmetry, and Figure 7 shows that, as overall length decreases, although the median stays around 0, i.e., symmetry, there are more and more extremely asymmetrical pieces. This pattern holds for both the entire assemblage (A and B) and the retouched pieces only (C and D), although the pattern is less strong there, partly because some of the least asymmetrical pieces are the large, unretouched ones. These patterns strongly suggest that the tools get resharpened in the haft *with a preference for one edge or another*. This is inconsistent with projectile use, but very indicative of use as knives or scrapers, i.e., edge-tools where the maintenance of a long edge is sought after during the reduction process.

PC 2 and PC 3, which represent relative elongation and, respectively, relative tang length and ‘pointedness,’ also exhibit size (length)-dependent patterns. The multiple regressions of PC 2 and PC 3 on length and tip length are significant, with a positive slope for PC 2 and a negative slope for PC 3 (although a relatively small proportion of the variance is explained, adjusted multiple R-squared = 0.21 and 0.25, for tip length, p<0.01). In visual terms, this translates to saying that long Aterian tanged tools are pointier and more elongated than short ones, and that they also have relatively shorter tangs. This is consistent with the hypothesis that Aterian tanged tools are resharpened in the haft, during which process they become duller and more rounded, again adding evidence against their use as projectile tips. As the tips get resharpened, the tangs remain in the haft, resulting in the more reduced pieces having relatively larger tangs. This is confirmed by running a regression of the logged tang length on the tip length, which documents the allometric growth of the tang in relation to the tip of the pieces (slope of the major axis regression: *b* = 1.23, significantly different from isometry (*b* = 1), p<0.05, confidence interval = (1.05, 1.48)).

**Figure 8:**
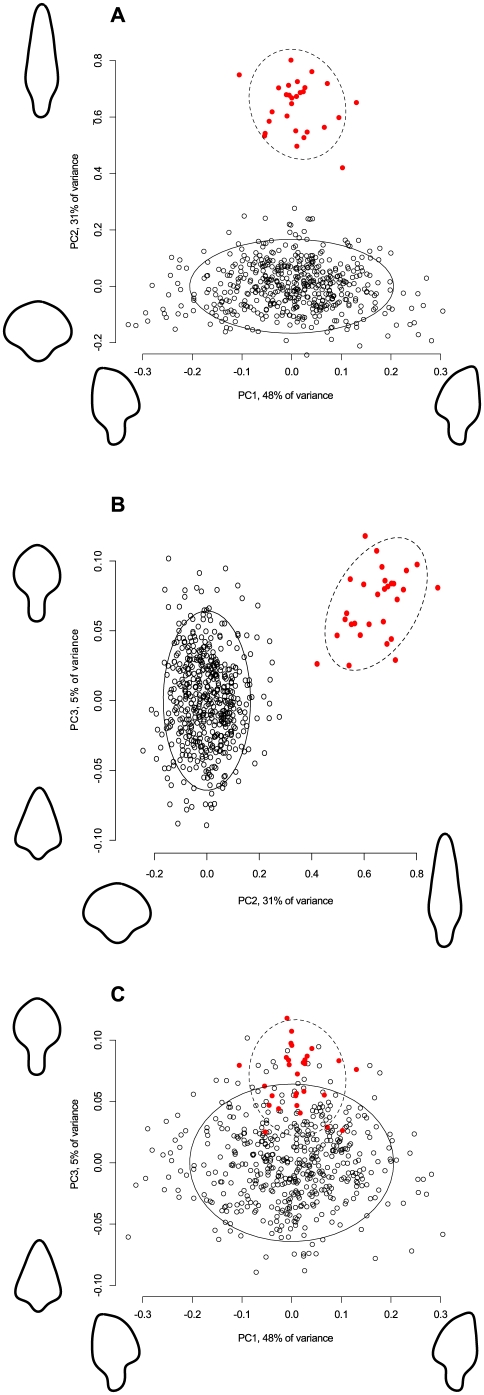
Graph showing the comparison of the Stellmoor and Aterian material on pairwise-drawn axes of the first three principal components of shape variation. The outlines on each axis represent extreme shapes along each component (see Method for an explanation). The ellipses represent 95% confidence intervals around the centroid of each data cluster.

**Figure 9:**
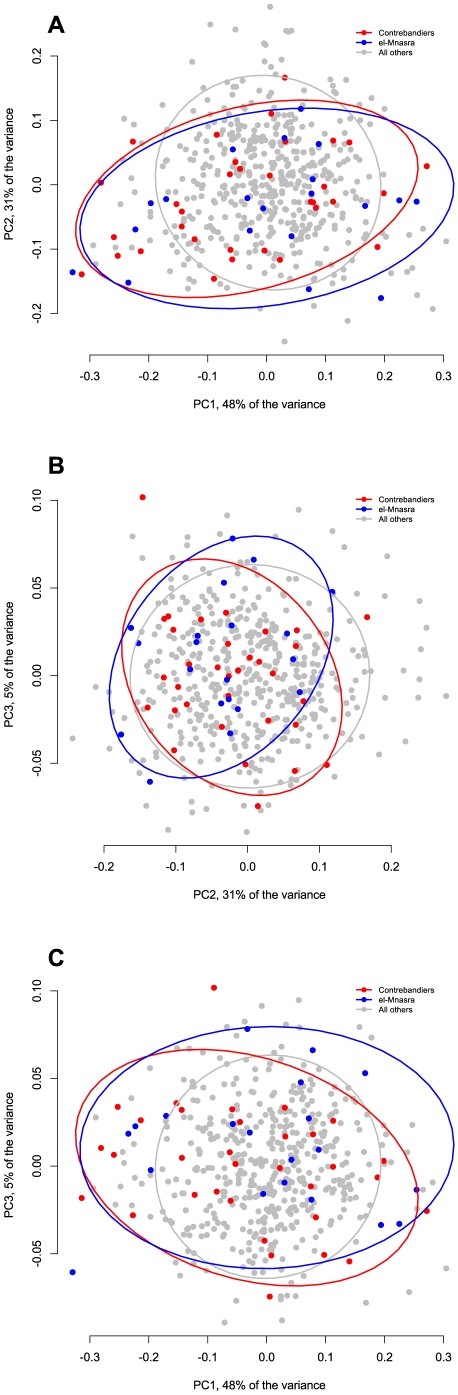
Graph showing the variation of the two main samples from recently excavated sites (El-Mnasra and Contrebandiers) in comparison with the rest of the Aterian sample. The ellipses represent 95% confidence intervals around the centroid of each data cluster.

In order to see how the shape variation in Aterian tanged tools compares with that found in tanged tools whose function as projectile tips is relatively well-known, the data from 29 complete projectile points from the North German Final Paleolithic site of Stellmoor were projected on the principal components of the Aterian shape variation. These points are known from association with wooden arrow shafts to be real flying projectile points, and their variation is considered to be constrained by both hafting and ballistic factors. The results show that the shape variation in the Stellmoor points is differently structured from that found in the Aterian. The Final Paleolithic arrowheads can be distinguished best along PCs 1 and 2 (Figure 8), because they are much more elongated and less variable with respect to side-to-side asymmetry than the Aterian pieces. This is not only a matter of sample size, as the two recently-excavated collections of El-Mnasra (n = 20) and Contrebandiers (n = 33), whose distribution within the PC-space is shown in Figure 9, show much more variation than the Stellmoor sample. These patterns serve to illustrate the way that Aterian tools, although similar in shape to later period arrowheads, are retooled unlike projectile points.

## Discussion

Several lines of evidence point in the direction of progressive resharpening of Aterian tools in the same manner as edge-tools such as scrapers and cutting-tools. This does not per se rule out an initial use for some Aterian pieces as weapon tips, because the ultimate use of each individual tool must be determined by the examination of use-traces, and because each episode of retouch likely wipes out previous uses of the tool. However, the data presented here make a strong case for the claim that, in general, these tools were probably hafted and used repeatedly for tasks that resulted in the need to rejuvenate *edges* rather than *point-tips*. The comparison between excavated, mostly cave contexts, and surface sites reveals that they both contain similar reduction trajectories and shape variabilities of tanged tools. This indicates that the functional emphasis on the tools was similar during their use-life in the landscape and at the repeated-occupation sites, which contradicts the expectations of breakage and repair patterns associated with a use as projectile tips [62], [63].

Regarding the behavioral implications of this result, it must first be stressed that all hominins were accomplished hunters both in Africa and elsewhere long before the appearance of the earliest Aterian tanged tools, and that killing from a distance using flying projectiles may not have conferred the advantages that are usually assumed. The faunal assemblages associated with Aterian occupations do not include large, dangerous prey animals, as do those encountered by Neandertals in Europe (e.g., reindeer and bison, see [4], [5]) or South African modern humans (buffalo and bushpigs, see [64]–[66]). Instead, these are dominated by gazelles [67]–[69], posing an entirely different kind of problem to potential hunters than avoiding direct contact with the hunted animal, which has been proposed as the cause of rodeo-rider-like injuries in Neandertals [70].

On the other hand, the same patterns in Aterian tool shape changes are consistent with their use in conjunction with presumably wooden shafts, resulting in a combination of different raw materials into a single, more complex tool. The evidence for an early invention and increasing prevalence of hafted tools is mounting, both in Europe, among Neandertals (e.g., [71], [72]) and in Africa, among anatomically modern humans ([73], [74]). In light of this evidence, it is perhaps more appropriate to treat the ‘Aterian question’ in terms of an early innovation in hafting, rather than in projectile use, or, indeed, in weapon use at all. And if that is the case, we must ask what prompted the invention of the hafting insert. It could be speculated that the invention of this distinguishing feature of the Aterian, the tang, was associated with a move into increasingly arid zones of the Sahara [19], [20], [75], where the lack of resin-bearing trees could have created the need for a hafting insert adapted for use with bindings (but see [73] for evidence of an increase in the use of resin for hafting in southern Egypt in the Upper Pleistocene and [76], [77] for an ethnographic account of spear-hafting using resin in the Australian desert). It is as yet unclear if gum-yielding plants would have been available in the more arid zones of North Africa 100 thousand years ago, but it makes sense that a tang allows for an easier hafting using leather bindings, since it provides a less sharp and more regular surface to wrap around. Either way, this hypothesis is one which can only be tested through residue and usewear analysis (including experiments), combined with paleoenvironmental studies, for all of which collections with a better quality of preservation and curation are necessary. Further, the paucity of excavated desert contexts limits our understanding of the extent and nature of the Aterian settlement of the truly arid zones of the Sahara to the level of mere presence or absence, a greater diversity of excavated sites being needed to warrant secure behavioral conclusions. Recent research in the arid zones of Australia [78] has shown that geomorphic and taphonomic biases can distort behavioral interpretations, especially with respect to the diversity and complexity of material culture. Fortunately, given the revived interest in the Aterian, we can expect the quality of the data to be improved soon by freshly excavated Aterian assemblages.

It is thus possible that hafting was practiced on both sides of the Mediterranean Sea, but in different ways. Although some of the differences in technological innovation between archaic and modern humans that we observe at the continental and species level may be due to cognitive differences or to demographic factors influencing the spread and accumulation of information [79], [80], we must not forget the essentially functional character of toolkits. Especially when comparing and evaluating technologies at very large scales, functional responses to specific technological problems (such as prey size and behavior [1] or increased risk associated with prey frequency and ease of hunting (e.g., [81]–[84])) may trump other factors. Even if the ultimate cause underpinning technological change is a large-scale environmental phenomenon, such as a rapid cooling event, or the aridity of a newly-colonized area, we can understand these associated changes only by unraveling the constraints imposed on toolkits by the subjects of the actions for which the tools themselves were used. Thus, perhaps the better question to answer regarding the Aterian might not be if it represents the earliest hunting weapons technology, but rather, in what way it arose out of new challenges posed by the environments that characterized North Africa since MIS 5, and how it adapted during the almost 100 thousand years of occupation of this region.

## Materials and Methods

### Method

All hafted stone tools reveal uneven resharpening patterns, because tools that break or become dull are resharpened in the haft, meaning that the active part of the tools are reworked, whereas the tangs are often spared modification and are discarded in largely original form. When a large enough sample is used, this time-series can be re-created by investigating the directionality of the shape changes using some measure of size as a proxy for time. The method is theoretically closest to ontogenetic scaling in biology, and has been recently used to determine approximate function in edge-tools European Paleolithic assemblages [85]–[87], as well as in Patagonia [88].

For quantifying shape, Elliptical Fourier Analysis was used to parametrize tool outlines (EFA[89]–[91]), and the data were subsequently reduced in dimension through Principal Components Analysis (PCA). Contour data for each specimen were automatically obtained from digital photographs of the artifacts, using a combination of scripts written by the author for Fiji (ImageJ, [92]) and R [93] (in collaboration with Shannon McPherron), and which are available at https://sites.google.com/site/raduiovita/morphometrics-software. Specimens were oriented with the dorsal side up and the tang facing the left of the photo, and outlines were then translated with the centroid at the origin and size-standardized by dividing the outline coordinates by the square root of their area before the EFA (see [85], [86]). Additionally, the coefficients were rotation-standardized using the orientation of the best-fitting ellipse as in [90], but without dividing by the length of the semi-major axis, since size standardization by area was preferred. This is because the variation in elongation, captured by the coefficients of the first harmonic, is interesting in a study of shape-change along a hypothesized longitudinal axis of reduction. For the statistical analysis only the first 11 harmonics (44 EF coefficients) of the parametrization were retained, as they captured more than 99% of the cumulative harmonic power (the halved squared sum of the harmonic coefficients) and 98% of the variance in the sample (for an implementation see [87], [94]). Finally, the graphical representation of the extreme shapes is obtained by adding to the mean shape the extreme (maximum and minimum) scores along each component, multiplied by the corresponding eigenvector. The coordinates are then obtained via the inverse Fourier function.

### Sampling

There are very few collections of Aterian tanged tools obtained through controlled modern excavations that are available for study, and these are often quite small. On the other hand, museum collections from excavations and surface collection in the beginning of the 20th century offer large numbers of complete tools that provide a big enough sample to allow a modern morphometric investigation of the shape changes during the process of retooling. In order to capture the outlines satisfactorily (95% of power/variance), at least 11 harmonics, and hence, at least 44 coefficients of the elliptical Fourier expansion are needed – resulting in the need for samples that contain at least 50 specimens or more. In order to increase the sample size, this study combines two complete samples from modern excavations (Contrebandiers and el-Mnasra) with those from old excavations (Tit Mellil, Oued Djouf el-Djemel, el-Oubira, Bir-Chaacha, Bir el-Ater, Koudiat Bou Gherara, Grotte du Polygone d'Eckmuhl) and surface collections from the Musée de l'Homme in Paris (REDA/RDAC (Ouargla), Beni-Abbes, Oum-el-Ksi, Nord-Azraza, Erg Chech, [Erg] Jmeyha, and Djouf Djoudder) (see Table 1).

**Figure 10:**
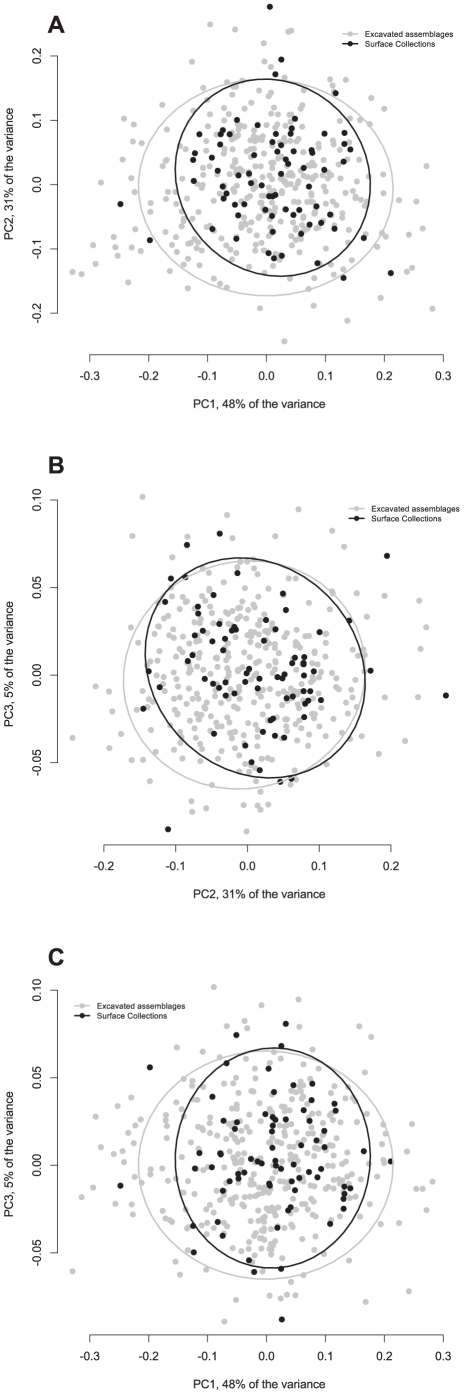
Graph showing the variation of the samples from all excavated sites (see **Table 1**) in comparison with the rest of the Aterian sample. The ellipses represent 95% confidence intervals around the centroid of each data cluster.

When using material from old excavations and surface collections, one must perform a cost-benefit analysis before proceeding, since subjective biases have been shown to influence the characterization of a lithic industry before, with big and ‘beautiful’ pieces being favored by excavators or collectors (e.g., [95]). An examination of Figure 10 shows that the variability present in the excavated collections is greater than or equal to that present in the surface collections, and is distributed in the same way along the first three principal components. Similarly, the two modern sites of Contrebandiers and El-Mnasra exhibit the same pattern as the rest of the pieces (see Figure 9), indicating that, by increasing the sample number with early 20th century excavated and surface-collected assemblages does not artificially change the results. This is actually not surprising, since the single criterion for collection was the presence of a tang, which is easy to identify. However, it also implies that there is no functional difference between the pieces that were brought home and were subsequently found in excavation, and those that were abandoned on the landscape. This is a crucial difference, because it is possible that the original function can only be seen on the pieces that were brought out for specific tasks in the landscape.

**Figure 11:**
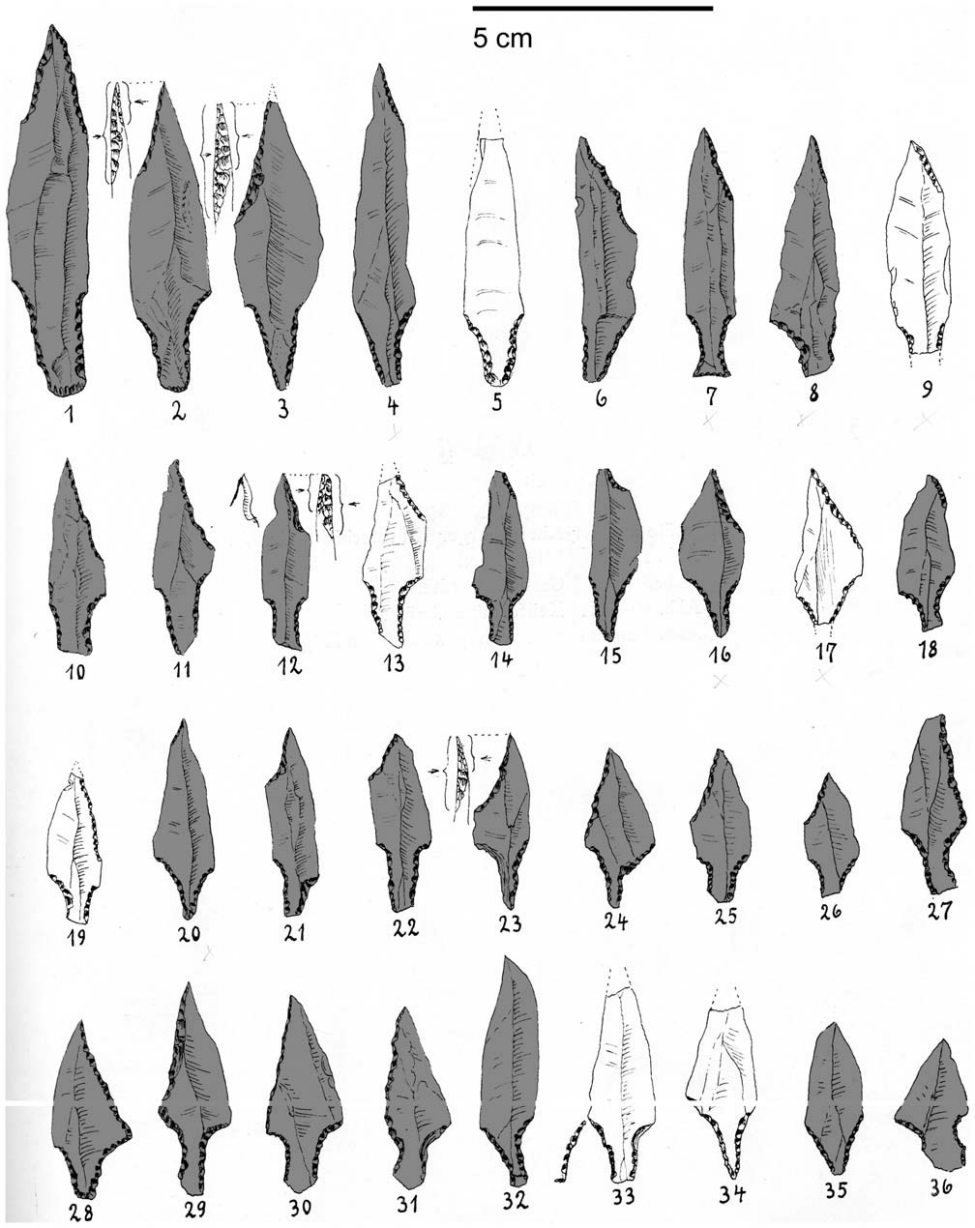
The 29 Stellmoor points chosen for analysis, in a modified version of Rust's 1943 Plate 46. The greyed specimens were selected for inclusion into this study.

The reason for choosing the Stellmoor material as a comparative standard is that this particular set of tanged points were found in the Stellmoor pond (44 specimens in total, out of which 29 were deemed complete enough for a shape analysis), in the same context as several wooden arrow shafts, with two tang fragments having been found within the shafts themselves [96]. Before the 1940s, some 500 more tanged points were found on the hill above the pond, which is the eponymous site for the Ahrensburgian culture [97], but the association of the Plate 46 points with the arrow shafts was considered to provide for a better comparative material. While the comparison with known flying projectiles of a similar general outline morphology from several tens of thousands of years later may seem unfair, it allows for the setting of a standard which is still anchored in a hunter-gatherer Paleolithic way of life, free of some of the concerns that affect the use of ethnographic standards in stone tool research. The outlines were obtained from the drawings in Plate 46 of Rust's monograph (see Figure 11) via the same techniques used for the photographs of the Aterian material.
